# Key Challenges in Plant Microbiome Research in the Next Decade

**DOI:** 10.3390/microorganisms13112546

**Published:** 2025-11-07

**Authors:** Ayomide Emmanuel Fadiji, Adegboyega Adeniji, Adedayo Ayodeji Lanrewaju, Afeez Adesina Adedayo, Chinenyenwa Fortune Chukwuneme, Blessing Chidinma Nwachukwu, Joshua Aderibigbe, Iyabo Olunike Omomowo

**Affiliations:** 1 Hawkesbury Institute for the Environment, Western Sydney University, Penrith, NSW 2753, Australia; 2 Food Security and Safety Focus Area, Faculty of Natural and Agricultural Sciences, North-West University, Private Bag X2046, Mmabatho 2735, South Africa; 3 State Key Laboratory for Biology of Plant Diseases and Insect Pests, Institute of Plant Protection, Chinese Academy of Agricultural Sciences, No. 2 Yuanmingyuan West Road, Beijing 100193, China; 4 Department of Biotechnology and Food Science, Faculty of Applied Science, Durban University of Technology, P.O. Box 1334, Durban 4000, South Africa; 5 Department of Biological Sciences, Western Illinois University, University Circle, Macomb, IL 61455-4454, USA; 6 Department of Life and Consumer Sciences, College of Agriculture and Environmental Sciences, University of South Africa, Rooderpoort 1710, South Africa; 7 School of Molecular and Cell Biology, Faculty of Science, University of the Witwatersrand, Private Bag 3, Johannesburg 2050, South Africa; 8 Department of Chemical Engineering, University of Abuja, Abuja 900105, Nigeria; 9 Department of Pure and Applied Biology, Ladoke Akintola University of Technology, Ogbomoso 210214, Nigeria

**Keywords:** sustainable agriculture, microbial complexity, global food security, environmental resilience, multi-omics integration

## Abstract

The plant microbiome is pivotal to sustainable agriculture and global food security, yet some challenges hinder fully harnessing it for field-scale impact. These challenges span measurement and integration, ecological predictability and translation across environments and seasons. Key obstacles include technical challenges, notably overcoming the limits of current sequencing for low-abundance taxa and whole-community coverage, integrating multi-omics data to uncover functional traits, addressing spatiotemporal variability in microbial dynamics, deciphering the interplay between plant genotypes and microbial communities, and enforcing standardized controls, metadata, depth targets and reproducible workflows. The rise of synthetic biology, omics tools, and artificial intelligence offers promising avenues for engineering plant–microbe interactions, yet their adoption requires regulatory, ethical, and scalability issues alongside clear economic viability for end-users and explicit accounting for evolutionary dynamics, including microbial adaptation and horizontal gene transfer to ensure durability. Furthermore, there is a need to translate research findings into field-ready applications that are validated across various soils, genotypes, and climates, while ensuring that advances benefit diverse regions through global, interdisciplinary collaboration, fair access, and benefit-sharing. Therefore, this review synthesizes current barriers and promising experimental and computational strategies to advance plant microbiome research. Consequently, a roadmap for fostering resilient, climate-smart, and resource-efficient agricultural systems focused on benchmarked, field-validated workflows is proposed.

## 1. Introduction

The plant microbiome, composed of diverse microbial consortia inhabiting plant roots, foliage, and internal tissues, plays an essential role in supporting plant health, enhancing resilience, and boosting productivity [1]. Recent developments in high-throughput sequencing and multi-omics platforms have yielded unprecedented insights into these microbial communities and their dynamic interactions with plant hosts, uncovering a spectrum of potentially advantageous traits that could revolutionize modern agriculture [2]. These benefits range from improved nutrient acquisition and resistance to biotic and abiotic stresses to enhanced crop yield and quality [3]. However, despite these advances, the intricate nature of plant–microbiome associations presents significant hurdles that need to be overcome to fully leverage these advantages in the coming decade [4]. Those challenges include technical constraints in sequencing technologies with standardization and reproducibility; data integration and analysis; the limited functional characterization of microbiome members; host specificity and environmental context dependence of assemblies; achieving stability, resilience, and predictability of engineered microbiomes; co-evolution and domestication effects on plant–microbe interactions; translating research into field applications; regulatory and ethical considerations; and the economic viability of microbiome-based interventions.

A major obstacle in plant microbiome research is understanding the specificity and functionality of plant–microbe interactions within diverse environmental contexts. Unlike laboratory-controlled experiments, real-world conditions expose plants and their associated microbiomes to highly variable environmental factors, including soil characteristics, climate, and seasonal changes [5]. Such variables influence microbial community composition and functionality, complicating efforts to predict and manage plant–microbiome interactions consistently across different agricultural settings [6,7]. Additionally, the plant microbiome’s dynamic nature means that microbes may change roles or lose efficacy over time, further complicating our understanding of how these communities support plant vigor and yield [1].

Furthermore, methodological challenges also present obstacles to advancing plant microbiome research. For example, extracting high-quality, reproducible data from microbial communities requires careful consideration of factors like sampling techniques, DNA extraction methods, and bioinformatics processing pipelines [8]. These technical issues can lead to significant discrepancies in data, hindering comparative analyses and long-term studies across different plant species and environments [9]. Recent studies have also highlighted the need for standardized protocols and innovative bioinformatic approaches to manage and interpret the vast amounts of data generated from plant microbiome studies effectively [10]. Without a consensus on these methodological standards, the field faces significant limitations in reproducibility and scalability. Thus, two technical priorities: sequencing technologies with standardization and reproducibility, and multi-omics data integration and analysis as enablers of comparable, decision-ready evidence were highlighted.

Ecological and evolutionary aspects of the plant microbiome add another layer of complexity. The stability and resilience of microbial communities within plants are influenced by ecological competition, mutualistic interactions, and evolutionary adaptation [11,12]. Gaining insights into these ecological processes is essential for anticipating how microbial communities will adapt to shifting environmental factors and agronomic interventions, including fertilizer or pesticide use, which may unintentionally disrupt microbial assemblages in ways that compromise plant health [2]. Efforts to engineer stable, beneficial microbial consortia must account for these ecological interactions to ensure that introduced microbes can successfully establish and perform desired functions in real-world conditions [13]. To this end, three biological challenges, the limited functional characterization of community members, achieving stability, resilience, and predictability in engineered microbiomes, and understanding co-evolution and domestication effects that shape plant–microbe partnerships over time were examined.

**Figure 1:**
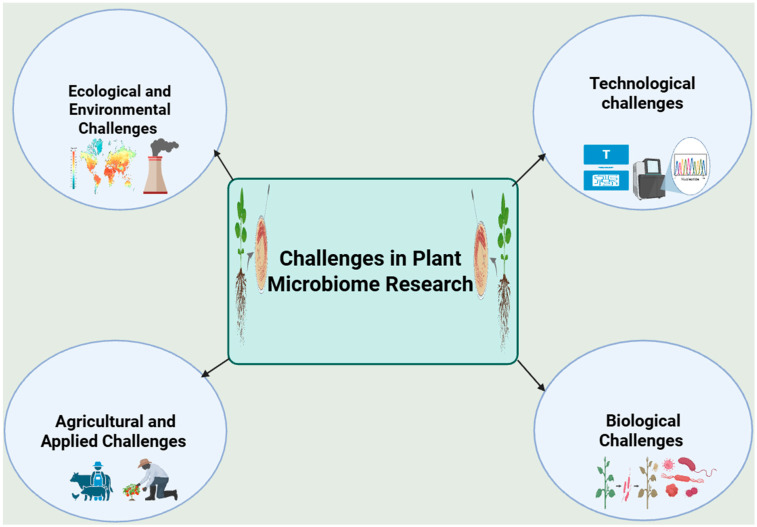
Key challenges of plant microbiomes in the coming decade.

Also, ethical, regulatory, and practical considerations are emerging as the field moves toward the commercial application of microbiome-based technologies in agriculture [14]. As researchers develop microbial inoculants and other bio-based interventions, questions regarding biosafety, intellectual property, and long-term ecological impact must be addressed to ensure responsible implementation. The need for a supportive regulatory framework that facilitates innovation while protecting ecosystems is becoming increasingly urgent as microbiome-based technologies approach widespread use [15,16]. Therefore, this review explores critical, scientific, technical, and ecological challenges limiting progress in plant microbiome research to achieve agricultural sustainability in the coming decade and outlines future directions to address these gaps. Consequently, two applied constraints, translation to field applications and economic viability coupled with regulatory and ethical considerations that govern deployment were assessed (Figure 1). Furthermore, it examines emerging insights and interdisciplinary strategies in a bid to contribute to a more comprehensive understanding of plant–microbe interactions and to facilitate the development of sustainable agricultural practices over the next decade.

## 2. Technological Challenges in Plant Microbiome Research

### 2.1. Sequencing Technologies: Limitations, Standardization and Reproducibility

Advances in metagenomic shotgun sequencing, amplicon profiling, whole-genome sequencing (WGS), and long-read platforms have reshaped plant microbiome research by resolving community structure, functional potential, and population dynamics across compartments and gradients [17,18,19,20,21,22,23]. Shotgun metagenomics supports broad, hypothesis-flexible discovery, whereas amplicon approaches provide cost-efficient taxonomic profiling; WGS deepens strain-level analysis; and metatranscriptomics adds activity-aware insights.

Despite this progress, shotgun metagenomics in plant systems is often limited by high host DNA, complex assemblies in diverse rhizospheres, and incomplete reference coverage, especially for fungi. Purpose-built tools such as EukDetect and MiCoP improve mycobiome detection, but incomplete and uneven databases still constrain taxonomic and functional accuracy [24,25,26]. Long-read and synthetic long-read approaches (for example, PacBio Sequel II, ONT, LoopSeq) increase contiguity, enhance strain resolution and improve MAG recovery and functional annotation relative to short reads, although higher input requirements, costs and bioinformatic demands currently limit routine use [27,28,29,30].

Outcome variability is also driven by upstream protocols. DNA extraction kits and washing procedures can differentially lyse taxa or alter biomass recovery, biasing both diversity and function estimates; repeated washes improve retrieval of rare but ecologically important taxa [31,32]. Across platforms, short and long-read data may yield similar community-level profiles, yet long reads more often resolve strains, novel taxa, and more complete MAGs. Downstream accuracy depends as much on reference choice as on algorithm: pairing high-quality databases such as SILVA or RefSeq paired with classifiers such as PathoScope or Kraken2 can outperform 16S-specific pipelines at genus and species levels [23,24].

Field heterogeneity complicates comparability across sites by altering microbial composition and activity. Soil moisture and related factors can confound cross-site inference, and quantitative SIP and other stable-isotope approaches link function to taxa and motivate harmonized sampling, host-depletion, depth targets, and transparent reporting of extraction kits, primer sets, databases, and classifier versions [33,34]. Combining these technological and standardization issues within a single framework clarifies where uncertainty enters the pipeline and how best-practice reporting can improve reproducibility across plant microbiome studies. Therefore, there is a need for field trial workflow that links host-aware sampling, suitable sequencing, and database-explicit analysis with clear depth targets and reporting and then validates these steps across plant organs and moisture conditions. A coordinated field benchmark would quantify gains in taxonomic, functional, and MAG recovery and improve comparability across studies.

### 2.2. Data Integration and Analysis

Combining multiple omics approaches including genomics, transcriptomics, metabolomics, and proteomics) is essential for providing a holistic perspective on plant microbiomes [35,36]. However, this integration poses considerable challenges. Each omics layer generates distinct types of data that vary in resolution, complexity, and scale, complicating efforts to synthesize them into coherent biological insights [37]. Additionally, Kumar et al. [37], emphasized the significance of integrating transcriptomics and proteomics data to decipher complex gene regulation mechanisms. Breakthroughs in high-throughput RNA sequencing and mass spectrometry (MS) have rendered these methods essential for studying global gene regulation. The integration of these datasets, coupled with improvements in big-data analysis, has provided insights into genome annotation, RNA-protein interactions, gene regulation, disease markers, and drug target identification. Also, various computational tools and approaches for integrative analysis were explored, highlighting that combining genomic data (16S rRNA sequencing) with transcriptomic (gene expression), metagenomic shotgun sequencing and metabolomic (metabolite profiles) data can be difficult due to the dynamic nature of plant–microbe interactions. Integrated repositories commonly used for multi-omics studies include MGnify microbiome resource [38], the Department of Energy (DOE) National Microbiome Data Collaborative [39], Integrated Microbial Genomes and Microbiomes (IMG/M) [40], and the DOE Systems Biology Knowledgebase (KBase) for genomes and metagenomes [41]; MetaboLights [42] and the Metabolomics Workbench for metabolite datasets [43]; Global Natural Products Social molecular networking (GNPS) with the Mass Spectrometry Interactive Virtual Environment (GNPS/MassIVE) for MS/MS molecular networking [44]; and PRoteomics IDEntifications Database (PRIDE) for proteomics [45].

Computational models capable of integrating multi-omics datasets remain in the early stages of development, and existing tools often struggle with scalability and complexity. For instance, the Microbiome Machine Learning Framework (MiMe) like examPle and others, are designed to integrate genomic, transcriptomic, and metabolomic data to provide a more holistic view of plant–microbe interactions, but requires continuous refinement to handle large and diverse datasets accurately [17,46]. Advances in bioinformatics platforms, such as tidyomics offer promising solutions by streamlining omics data analysis and facilitating cross-disciplinary collaboration [47]. Examples of AI-driven or statistics integration pipelines now in routine use include the Quantitative Insights Into Microbial Ecology 2 (QIIME 2) sample-classifier plugin for supervised learning [48], Statistical Inference of Associations between Microbial Communities and host phenotypes (SIAMCAT) for cross-validated association modeling [49], Songbird for compositional differential ranking [50], mmvec for learning microbe–metabolite embeddings [51], and Model-based Integration of Metabolite Observations and Species Abundances 2 (MIMOSA2) for linking metagenomes to metabolite shifts via metabolic modeling [52].

However, the challenge remains in developing universally applicable models that can handle data from multiple studies, especially when environmental conditions vary. The integration of multi-omics datasets is fundamental for decoding plant microbiomes; however, significant challenges remain in overcoming the variability in data resolution, complexity, and scale. Moreover, recent innovations in computational modeling and bioinformatics tools such as MiMe and tidyomics hold great promise. However, the creation of robust, universally adaptable modeling frameworks remains ongoing, thus requiring continuous advancement. Accordingly, the ongoing optimization of these systems is vital for enhancing both the precision and comprehensiveness of analyses focused on plant–microbe interactions across heterogeneous environmental contexts.

Summarily, a persistent bottleneck is the combination of low microbial biomass, high host DNA, incomplete fungal reference coverage, and analytical complexity, which together limit taxonomic and functional resolution in plant-associated datasets. A practical way forward is to pair host-depletion or capture enrichment with long-read or hybrid assemblies to improve MAG and strain recovery, use fungal-aware classifiers such as EukDetect or MiCoP with curated databases, and report depth targets, database versions, and pipeline steps transparently to support replication. Fungal taxonomic identification and abundance estimation are well conducted using tools such as EukDetect and MiCoP. Long-read platforms, including PacBio and Oxford Nanopore, together with synthetic long-read approaches, enable full-length profiling and more contiguous assemblies, yielding species-level improvements.

## 3. Ecological and Environmental Challenges

### 3.1. Understanding the Dynamic Nature of Plant–Microbiome Interactions

The increasing recognition of the interconnectedness between plants and their microbiomes has introduced new challenges in the fields of biology and evolutionary science. Microbial communities are now widely associated with host development, ecological adaptation, and evolutionary processes [53,54,55,56,57]. In the plant holobiont, the host and its microbiota form a dynamic, interdependent system that spans the rhizosphere, endosphere, phyllosphere, and spermosphere, with each compartment supporting distinct communities shaped by pH, oxygen availability, and nutrient levels [58,59,60]. Complex multi-kingdom processes influencing organism-domain interactions make it difficult to apply SynCom stability, persistence, and function in diverse, non-sterile soils to enhance real-world translational value. Across interaction modes that include mutualism, commensalism, competition, and antagonism, roots release diverse compounds that recruit beneficial microbes and deter pathogens, while microbes modulate hormone signaling, nutrient uptake, and root architecture [61,62].

Numerous investigations have aimed to define a fundamental microbiota, referring to the microbial taxa recurrently found across most plant species [63,64]. Establishing this core microbiome allows us to recognize a collection of stable taxa that are more likely to affect plant phenotypes [65]. The core microbiome concept needs further exploration into the ecological principles at play, including identifying keystone species [66], and the concept of functional redundancy [67].

The keystone species framework [66] proposes that the ecological influence of a single taxon may be disproportionately large relative to its abundance. In contrast, the functional redundancy model argues that multiple taxa with overlapping functions can ensure continuity of ecosystem functions, thereby offering resilience to perturbations [67]. Loss of a keystone taxon typically leads to a decline in ecological functionality, whereas redundancy implies that ecosystem processes can be sustained through the presence of functionally similar taxa. Furthermore, communities may exhibit functional complementarity as members with diverse roles contribute to the stability of the community [68]. Thus, shifts in the taxonomic composition of a community do not necessarily result in functional loss, as the outcome depends on the retention of functional attributes and complementary roles within the system. These theoretical constructs may also inform microbiome elasticity under compositional changes [69], providing a lens for interpreting core microbiome dynamics [70]. Yet, few studies experimentally validate the roles of core taxa, keystone microbes, or redundancy under changing environments. Future investigations should evaluate these ecological theories in plant–microbiome contexts using targeted knockouts, trait mapping, and long-term monitoring.

Another important variable that can influence the role of different taxa within a microbial community is the community’s stage of development or maturity. Carlström et al. [71] conducted a study using a leaf SynCom consisting of 62 native bacterial isolates, demonstrating that specific strains from defined taxa acted as keystone members. They found that both the order and timing of keystone species introduction within the phyllosphere community had significant effects on how various strains or functional groups interacted; a phenomenon known as priority effects. In the early stages of community formation, removal of certain taxa markedly impacted the community’s overall structure. However, once the community was established, eliminated strains or taxa could recolonize the phyllosphere, though they no longer exerted the same influence on community architecture [71]. The study also highlighted that the order in which microbial strains are introduced to the host plant shapes not only colonization success but also affects how individual strains contribute to phyllosphere and rhizosphere assembly [71,72]. However, priority effects are rarely accounted for in microbial inoculant design or microbiome-based interventions. Longitudinal studies should explore how historical contingencies shape microbiome resilience, succession, and plant response.

Additionally, niche competition has been recognized as a key driver shaping the structure of plant-associated microbial communities. Schäfer et al. [73] combined experimental approaches with genomic modeling to predict microbial interactions in the phyllosphere. Their findings indicated that carbon metabolism, niche differentiation, and cross-feeding strategies are essential in governing microbial dynamics in oligotrophic phyllosphere environments. However, microbe–microbe interactions in spatially structured niches like the phyllosphere and rhizosphere are influenced by their highly heterogeneous spatial arrangements and unique physicochemical properties. Recently, Schlechter et al. [74] studied resource competition using a model based on a phyllosphere epiphyte (Pantoea eucalypti 299R) and six additional strains from two microbial phyla. They concluded that metabolic redundancy-driven competition is more pronounced in microbiota properties from controlled environments to field uniform microhabitats than in the spatially variable phyllosphere. Schlechter et al. [74] showed that macro-scale observations at the leaf level may fail to reflect fine-scale microbial competition patterns. Moreover, they proposed that competitive outcomes are not solely dependent on metabolic traits but also on features such as bacterial motility and the biosynthesis of antagonistic molecules. Getzke et al. [75] further supported this by demonstrating that strain-specific exometabolite secretion affects binary competition in the *A. thaliana* rhizosphere, with root-associated microbes exhibiting greater inhibitory capacity than soil-dwelling counterparts. Despite these insights, many interactions remain inferred rather than demonstrated. There is a pressing need to validate predicted competition or cooperation patterns using direct perturbation assays and spatially resolved metabolomics.

Furthermore, the importance of plant ontogeny in directing the development of plant-associated microbiomes has been increasingly recognized. Studies show that microbial communities linked to different plant organs undergo notable compositional shifts throughout the plant’s growth cycle [76,77]. Edwards et al. [77] examined the life cycle of rice grown under field conditions across diverse temporal and spatial gradients. They concluded that root microbiome composition is shaped not only by plant age but also by developmental stage. Developmentally correlated shifts in root microbial structure were associated with the transition of plants from juvenile to reproductive phases. Chen et al. [78] further demonstrated that root-exuded organic carbon fluctuates with plant developmental stage, impacting bacterial community dynamics more strongly than it does fungal communities. Moreover, Beilsmith et al. [79] showed that plant tissue type and developmental phase have a greater effect on microbial assemblages than geographic location. They observed that phyllosphere communities became increasingly distinct as plants matured, underlining the crucial role of host development in modulating plant microbiota. Additionally, the leaf surface is instrumental in shaping the phyllosphere microbiota, serving as a platform for diverse plant–microbe interactions [80]. Together, these observations reinforce microbial ecological interactions and host development jointly shaped plant phenotypes. The development and characteristics of the plant significantly influence the population dynamics of its microbiota. Therefore, further investigations into the dynamics of plant microbiomes ought to integrate ecological dimensions, including the interplay of competition and collaboration among microbial entities, as well as the influence of the plant’s context, encompassing developmental stages, stress responses, and ecotypes. To move from description to mechanism in real agricultural systems, studies should combine ecological network analysis, spatiotemporal meta-omics, and high-resolution imaging to identify which taxa interact, what they do, and how those actions alter plant performance [58,60,61,62,81].

### 3.2. Influence of Abiotic Factors on Plant Microbiome Composition and Function

Microbial communities underpin soil fertility and health, while environmental factors like drought, submergence and agrochemicals alter soil chemistry and biology. Soil physicochemical parameters govern gas diffusion and adsorption to soil particles and microbial cell surfaces. The interconnections among factors such as soil porosity, pH and organic carbon, which are physicochemical qualities, shape microbial density and activity. Soil physical chemistry affects microbial populations, and abiotic variables such as climate change and edaphic and management factors (including parent material, agricultural practices and land use) also impact it. The interactive and frequently non-linear responses of soil microbiomes to various abiotic factors are inadequately characterized, particularly in the context of future climate variability scenarios. Understanding these intricate interactions necessitates the development of high-resolution spatiotemporal models and integrative omics platforms to forecast microbiome resilience and plant outcomes.

#### 3.2.1. Soil pH

Soil pH is one of the strongest predictors of soil microbiome composition, which is influenced by metal toxicity, soil structure and texture, water supply and land use intensification. Beneficial soil microorganisms and plants commonly prefer near-neutral conditions (pH 6–7); departures from neutrality often shift microbial composition and activity [82]. Typically, soil pH affects fungal communities, but other environmental factors, such as physicochemical characteristics, also significantly influence their structure and dynamics. Fernández-Calviño and Bååth [83] found that modest pH shift reduces the total microbial biomass and select for pH-tolerant taxa. Intensive land use can affect soil structure by raising soil pH and reducing carbon concentration and water retention [84], with effects on microbial communities varying by land use type. Low diazotroph community diversity in acidic soils in alpine meadows implies that N_2_ fixation may be constrained [85]. Bacteria vary in soil acidity and alkalinity tolerance. *Azospirillum* density is relatively unaffected by soil pH; however, *Bradyrhizobium* communities often thrive in acidic soil and *Mesorhizobium* declines [85]. Alkali soils are often depleted in organic matter and nutrients, making them unsuitable for agriculture. Lower microbial activity contributes to poor performance in alkali soils. Jones et al. [86] and Mayerhofer et al. [87] report pH sensitivity among Acidobacteria: acidophilic subgroups (1–3) tend to increase as pH decreases, whereas others (4, 6, 7) are more abundant at higher pH; responses can be clade-specific. Although numerous correlative studies exist, the mechanistic understanding of the impact of pH-induced community shifts on plant health and microbe-mediated nutrient cycling is still incomplete.

Future research must combine long-term pH manipulation experiments with metatranscriptomic and metabolomic methods to clarify the causal pathways connecting microbial dynamics to plant phenotypes.

#### 3.2.2. Soil Temperature

Climate variation influences soil temperature, carbon dioxide (CO_2_), and precipitation patterns. Microorganisms are often categorized into three distinct groups: psychrophiles (≤15–20 °C optima), mesophiles (~20–45 °C), and thermophiles (≥50 °C). In temperate forests, soil microbes exhibit a notable relative abundance, with a temperature increase of 5 °C leading to a greater bacterial population relative to fungi [88]. The varying temperatures will influence the essential enzymes present in nitrogen-fixing bacteria. For instance, at temperatures near 5 °C, vanadium nitrogenase emerges as the most efficient enzyme utilized in the N_2_-fixation process. At elevated temperatures, approximately 30 °C, molybdenum nitrogenase exhibits enhanced efficacy owing to its superior affinity for N_2_ compared to vanadium nitrogenase. Elevated temperatures may also promote the proliferation of novel pathogenic strains at increased latitudes [89]. The progression of disease induced by *Verticilium dahliae* in olive cultivars is influenced by soil temperature. Elevated CO_2_ can upregulate salicylic acid (SA) signaling while dampening jasmonic acid (JA) signaling, which is crucial for enhancing resistance against *V. dahliae* invasion [89]. The influence of soil temperature on arbuscular mycorrhizal fungi (AMF) colonization varies across regions, contingent upon the differing carbon-to-nitrogen ratios. Frater et al. [90] noted that AMF colonization escalated with rising temperature and pH levels during experiments conducted in the Central United States. Conversely, Goicoechea [89] reported a decline in AMF colonization across the Mediterranean as temperatures increased. Jerbi et al. [91] and Frater, Borer, Fay, Jin, Knaeble, Seabloom, Sullivan, Wedin and Harpole [90] concur that warmer temperatures can enhance AMF colonization partly via increased root elongation. The thermal responses of microbial guilds have been documented; however, the effects of prolonged warming on mutualistic symbioses, pathogen suppression, and plant genotype-specific microbiomes are not well understood. Reciprocal transplant experiments, multi-year soil warming trials, and genotype–microbiome interaction studies are essential to predict microbiome-mediated plant responses to global warming.

#### 3.2.3. Soil Aeration

Soil hypoxia refers to a condition in which the soil experiences an oxygen deficiency, primarily from waterlogging [92]. Hypoxia induces stomatal closure in plant tissues, resulting in energy deficits and hindering the development of root systems, thereby diminishing their ability to uptake water and essential inorganic nutrients [93]. In aerobic organisms, the presence of oxygen inhibits the activity of nitrogenase. Consequently, diazotrophs use oxygen-protection strategies to sustain N_2_-fixation amidst the presence of oxygen including respiratory protection, heterocysts or hemoglobin-mediated O_2_ buffering, and extracellular polymeric substances that slow O_2_ diffusion [85]. Diazotrophs possess the capability to diminish oxygen levels through an increase in substrate consumption, subsequently enhancing respiration rates and resulting in reduced oxygen concentrations [85]. Root exudates can stimulate rhizosphere respiration, further reducing O_2_. Nevertheless, with sufficient carbon, diazotrophs can still fix N_2_ under relatively high O_2_. Energetically, assimilatory nitrate reduction is generally less costly than N_2_ fixation; diazotrophy is favored when combined nitrogen is scarce despite its high ATP demand. Actinobacteria predominantly thrive in aerobic conditions, necessitating oxygen for their metabolic processes. This characteristic accounts for their scarcity in floodplains [94]. Although microbial strategies for managing oxygen stress are well-documented, there is limited understanding of how changes in aeration regimes affect microbe–plant interactions, especially in waterlogged or compacted soil environments. Future research should integrate in situ O_2_ microsensors, spatial metabolite profiling, and diazotroph activity markers to delineate hypoxic micro-niches and anticipate functional changes in flooded agroecosystems.

#### 3.2.4. Soil Moisture

In many soils, moisture exerts a stronger short-term control on respiration than temperature [95]. The diversity of microbial communities in moist soils is notable; however, excessive water content can suppress microbial biomass. This effect is linked to reduced oxygen availability, which creates unfavorable conditions for aerobic organisms, including Gram-negative, Gram-positive bacteria, and mycorrhizal fungi. Soil moisture acts as a key determinant of soil biological activity [96]. Yet elevated moisture levels in soil environments pose a major constraint on aerobic microbial populations, as oxygen diffusion diminishes with increased water saturation. Consequently, microbial growth and activity decline, slowing the decomposition and mineralization of nitrogen and carbon. Under such conditions, the composition of microbial communities transitions toward anaerobic consortia, resembling communities found in aquatic systems. Cells retain sufficient water to sustain their metabolic processes and structural integrity by maintaining a more negative internal water potential than the surrounding matrix [97]. The long-term effects of varying moisture regimes on microbial community resilience, particularly in the context of seasonal drought and flood cycles, are not well quantified. Field experiments that incorporate soil moisture sensors, root exudate analyses, and microbial functional profiling are essential for predicting microbiome stability during extreme weather events.

### 3.3. Managing the Impact of Climate Change on Plant Microbiomes

**Figure 2:**
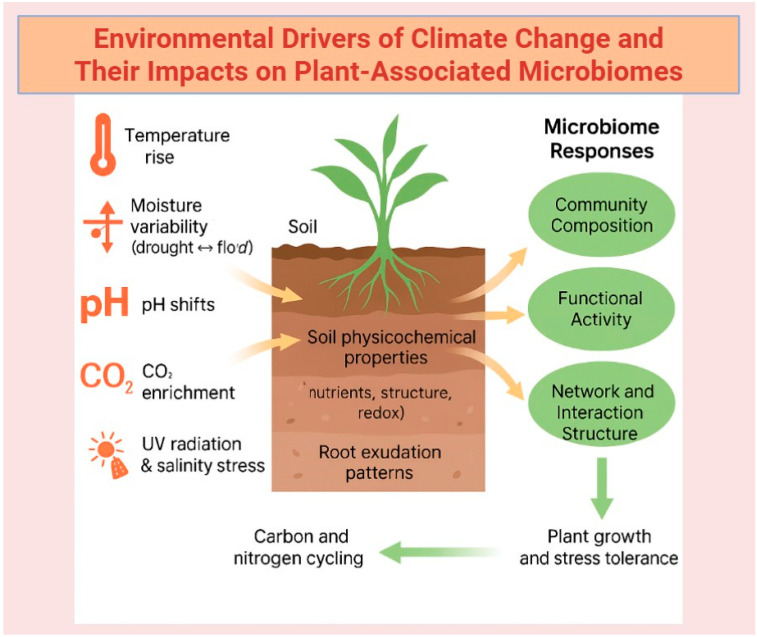
Environmental drivers of climate change shaping plant-associated microbiomes.

Climate change may significantly alter pathogen abundance and behavior, reshape host–pathogen dynamics, and facilitate the emergence of new pathogenic strains [98]. Figure 2 summarizes the different environmental drivers of climate change that shape plant-associated microbiomes. A significant fraction of various plant pathogens is anticipated to become more prevalent as global temperatures rise [99]. Compounding this issue, many traditional disease management strategies become less effective under elevated temperature regimes [100]. Concurrently, pathogens may evolve novel invasion strategies through alterations in their virulence mechanisms, potentially compromising the effectiveness of R gene-mediated plant resistance. In numerous plant pathosystems, interactions between higher temperatures and drought have been shown to weaken effector-triggered immunity (ETI), increasing disease susceptibility [101]. Much of the existing research on the influence of climate change on host–pathogen relationships rely on oversimplified models involving a single host plant interacting with one pathogen. However, under natural conditions, plants encounter a broad spectrum of potentially pathogenic microbes (pathobiota) [102]. The successful colonization of pathogens in these systems is largely determined by interactions of cooperation or competition between the pathobiota and the resident plant microbiome. Accordingly, this section explores how soil microorganisms exhibit community-level and physiological adaptations to environmental stressors driven by climate change.

#### 3.3.1. Elevated Carbon Dioxide (CO_2_)

Elevated atmospheric CO_2_ levels can boost plant carbon assimilation, shift carbon allocation patterns, and stimulate microbial activity in the rhizosphere [103,104,105]. Different plant species respond uniquely to rising CO_2_, thereby influencing carbon quantity and diversity released to the rhizosphere. For example, C_4_ plants, which fix carbon into a four-carbon compound, typically exhibit higher photosynthetic efficiency than C_3_ plants, which fix carbon into a three-carbon compound [96]. C_4_ plants are often more photosynthetically efficient under elevated CO_2_ and may allocate more carbon to root exudates, modifying microbial community composition in the rhizosphere [106,107]. This shift in belowground carbon flow has the potential to prime soil organic carbon (SOC) turnover by altering microbial decomposition dynamics [108]. Priming effects occur when new carbon inputs stimulate decomposition of existing SOC through the activation of microbial communities or introduction of labile substrates like root exudates and litter inputs, which may be intensified by elevated CO_2_. Meta-analyses and modeling approaches show that increased CO_2_ initially enhances photosynthesis and raises soil carbon inputs, but over time may also accelerate SOC decomposition [109,110]. This destabilization of carbon pools via microbial feedback loops adds complexity to soil carbon dynamics, especially in long-term soil carbon stabilization [111,112]. The difficulty in detecting changes in soil carbon stocks stems from the yet-to-be-understood fundamental biology governing soil organic matter decomposition [113]. A recent examination of priming effects in temperate and tropical forest soils revealed that the quantity of soil carbon released through priming did not correlate with the rates of soil respiration, attributed to variations in SOC turnover rates across the distinct soil biomes [104]. These findings suggest that priming is influenced by both the amount and quality of organic inputs, as well as microbial metabolic capacity. Overall, unraveling how elevated CO_2_, temperature, precipitation, and nutrient availability interact to influence microbial-mediated SOC turnover remains a critical research priority.

#### 3.3.2. Increased Temperature

The soil microbiome exhibits community-level and physiological shifts in response to elevated temperature, depending on the type of biome under investigation, for instance, forests versus grasslands. Warming has been shown to elicit contrasting effects on soil fungal populations across multiple boreal forest systems, resulting in either enhanced or diminished fungal biomass and activity; these variations are likely attributable to differences in soil moisture and/or vegetation at distinct locations [114,115]. The Harvard Forest Ecological Research Station Long Term Ecological Research site warmed soil by 5 °C above ambient temperature for 26 years to study the effects of extended soil warming on the temperate forest soil microbiome [116]. This long-term study found that microbial respiration and associated mechanisms acclimated in four phases: accelerated carbon loss via respiration, microbial community restructuring, and a transition toward a more diverse, oligotrophic microbial community exhibiting elevated soil respiration in heated environments compared to controls. This shift is accompanied by a decline in recalcitrant carbon pools, suggesting continued alterations in community structure [116]. Short-term soil respiration acclimation was attributed to reduced microbial biomass and thermal adaptation [117]. The physiological traits of microbial taxa must be quantified under in situ conditions, and recent isotopic methodologies enable estimation of microbial population turnover in field settings [118].

The intricate interplay of drought conditions, temperature increases, and the specific types of vegetation present fundamentally influences the capacity of microbial communities to withstand elevated temperatures. The Prairie Heating and CO_2_ Enrichment (PHACE) experiment, conducted on the grasslands of Wyoming, investigated the effects of 12 years of elevated CO_2_ alongside warming conditions [119]. Genes associated with carbon and nitrogen cycling exhibited enrichment when subjected to elevated CO_2_ levels, both independently and synergistically with warming conditions. Nonetheless, the process of nitrogen cycling was inhibited solely under warming conditions. The favorable response of the plant community, which led to an increase in biomass, further amplified the impact of changes in precipitation [120,121]. Although warming increased carbon input into soil and soil respiration, the associated carbon losses were largely offset by enhanced plant biomass. Together, these responses have the potential to weaken the positive feedback loop between climate warming and soil carbon depletion. While many climate projections predict a positive feedback loop between warming and soil carbon release via increased microbial respiration and reduced carbon storage [122,123], empirical evidence remains inconsistent across ecosystems. The relationship between warming, host plant genotype, and microbiome function, despite extensive research on thermal acclimation, remains largely unexplored. Future research should combine genome-resolved metagenomics with the assessment of plant traits across thermal gradients.

#### 3.3.3. Permafrost Thaw

Climate change is intensifying the seasonal thawing of the active layer, contributing to degradation of the underlying permafrost. As permafrost melts, enhanced water availability and microbial activity can elevate soil organic carbon (SOC) decomposition, thereby increasing CO_2_ and CH_4_ emissions [124]. Microbial activity is highly sensitive to permafrost moisture changes, particularly under thaw conditions. For example, CH_4_ generation is strongly influenced in thawed permafrost zones, modulated by topography, soil depth, and redox gradients [125,126,127]. Warming has been shown to reduce redox potential at permafrost interfaces, leading to higher methanogen abundance [128]. In contrast, intact permafrost zones may encourage iron reduction due to altered redox states. Moreover, iron concentration (Fe II) may directly shape microbial community structure in discontinuous permafrost soils [129]. Recent advances have elucidated how microbial communities respond to thaw events, particularly using molecular methods [125,126,127,130]. Metagenomic sequencing reveals that permafrost microbial consortia are distinct from those in the active layer and can shift rapidly during thaw [125,126,130]. These microbial assemblages vary across Arctic permafrost environments, with taxa like Actinobacteria increasing with permafrost depth, although species-specific trends exist [126,131]. To investigate microbial adaptation, metagenome-assembled genomes (MAGs) have been recovered from previously uncharacterized taxa [126,127]. These MAGs from recently thawed permafrost layers display key survival traits such as antifreeze proteins, heat shock proteins, DNA repair enzymes, and cryoprotectants [126].

The MAGs revealed how distinct soil microbiome members adapt to shifting nutrient availability during permafrost melt. For example, MAGs from a permafrost thaw gradient include genes for degrading plant polysaccharides, such as cellulases and xylanases [127], indicating their potential to digest plant-derived substrates. Another research found that MAGs with glucose metabolism genes increased after 4.5 years of 1 °C warming in the field [128]. Several MAGs are associated with methanogens, particularly in more humid environments where methanogenesis occurs. Nonetheless, a varied ability for CH_4_ oxidation has been uncovered in MAGs, indicating the presence of genetic mechanisms that facilitate the consumption of CH_4_ from thawing permafrost before its release into the atmosphere [132].

A noteworthy number of viral sequences have been discovered in Arctic metagenomes, many of which harbor auxiliary metabolic genes linked to plant polymer degradation and interactions with diverse microbial taxa participating in carbon cycling [133]. These viral communities vary along the permafrost thaw continuum, with a transition from ‘soil-like’ viruses in drier soils to ‘aquatic-like’ viruses in wetter zones [134], and are frequently associated with putative bacterial hosts, some of which are involved in soil organic matter decomposition. These observations suggest that both viruses and bacteria could substantially influence carbon turnover in thawing permafrost. Although limited data exist on fungal succession in permafrost, evidence suggests that certain taxa, including mycorrhizal fungi, may increase post-thaw [135]. One study reported that fungal community structures differ between permafrost rooting zones and nearby thawed bogs, with a post-thaw rise in potential saprotrophic and pathogenic fungi. These fungal community shifts are likely driven by parallel changes in plant functional traits resulting from warming [136]. Thus, more studies are needed to decipher fungal roles in permafrost systems and to model virus–host dynamics in carbon feedback loops.

#### 3.3.4. Drought

Climate change is anticipated to result in drought, a significant outcome within mesic grassland ecosystems. It is expected that the escalation of drought conditions will reduce microbial functions crucial for ecosystems’ sustainability [137,138]. As the moisture content of soil diminishes, the water present in soil pores decreases, leading to the formation of isolated resource patches; consequently, there is a reduction in the decomposition and respiration of soil organic carbon to CO_2_ [139]. The interplay of these factors produces responses that vary from diminished productivity in drier conditions to decreased carbon loss due to inhibited respiration [123,140].

Soil microorganisms exhibit diverse physiological mechanisms to cope with drought stress, including osmotic regulation, dormancy or reactivation, and the production of extracellular enzymes [141]. Microorganisms synthesize solutes (osmolytes) to maintain cellular turgor and endure diminished water matric potentials [139]. Nevertheless, the accumulation of osmolytes may prove excessively costly in severe desiccation conditions [142,143]. Soil microorganisms may endure in a desiccated condition, subsequently reviving and proliferating when moisture is reintroduced. An additional strategy includes the synthesis of extracellular polymeric substances (EPS) that help retain water under low matric potential [144]. Certain bacterial groups such as Actinobacteria and Bacilli exhibit exceptional drought tolerance, attributed to their efficient energy conservation, metabolic adaptability, and ability to enter dormancy in arid environments [145,146]. The costs associated with osmolyte production and the dynamics of microbial reactivation during repeated drought cycles are not well understood. Thus, future research should utilize time-series functional assays to analyze microbial legacy effects and adaptive capacity in response to varying water regimes.

#### 3.3.5. Increased Precipitation and Flooding

Increasing soil moisture leads to water-filled, anaerobic pores, enabling methanogenesis, denitrification, and release of CH_4_ and N_2_O. Changing precipitation patterns may cause differences in moisture and vegetation, leading to divergent microbial community responses; thus, metabolic models are necessary for realistic forecasts of future climatic situations [147]. Furthermore, the primary factors influencing CH_4_ concentrations in wetlands encompass soil temperature, water-table depth, and the composition of SOC [148]. Wetland regions, including peatlands, have the potential to shift from functioning as carbon sinks to becoming carbon sources in the future, thereby exacerbating existing warming trends. Nonetheless, the inundation of peatlands may impede the oxidative decomposition of soil organic carbon within the peat, leading to an overall increase in carbon sequestration.

Rising global sea levels are causing saltwater intrusion in coastal regions at a pace of 3.2 ± 0.4 mm per year [149]. Consequently, coastal soil ecosystems are vulnerable to saltwater intrusion, thereby introducing salt and sulfate, which changes the redox cycling dynamics and increases soil microbial biomass [150] and SOC mineralization, leading to increased CO_2_ production. Consequently, the ramifications suggest a prospective net escalation in greenhouse gas emissions due to heightened CO_2_ output as sea levels continue to ascend. Nonetheless, various coastal soils exhibit distinct responses to heightened salinity levels [151]. Extended periods of flooding resulted in a decline in microbial activity, attributed to resource depletion, illustrating a cyclical pattern of abundance and scarcity [152]. The response of microbiomes to concurrent changes in salinity, flooding, and nutrient levels is inadequately characterized; hence, there is a need to employ mesocosm experiments that integrate sea-level rise simulations with microbial trait mapping.

### 3.4. The Role of Horizontal Gene Transfer and Microbial Adaptation in Shaping Plant–Microbe Interactions

Mobile genetic elements (MGEs) are DNA-based entities that can move within or between cells, enabling genetic exchange and adaptation. Intracellular mobility refers to movement within a single genome, while intercellular mobility involves horizontal gene transfer across species or cells [153]. Examples of mobile genetic elements encompass plasmids, transposons, small RNAs, and prophages [153,154,155]. Prophages are bacteriophage-derived genetic elements that exist either episomally as plasmids or integrated into the bacterial chromosome [156,157]. Emerging research shows that phage DNA can also integrate into the host’s plasmids, forming phage–plasmid hybrid structures [158]. The presence of prophages often protects host bacteria from superinfection by related phages and enhances host survival through colonization in new ecological niches [159].

Horizontal gene transfer (HGT) from prokaryotes to plants is a prevalent phenomenon, notably exemplified by the integration of tumor-inducing genes from Agrobacterium into host plant genomes [160]. The virulence mechanism in Agrobacterium is triggered by phenolic compounds released from damaged plant tissues, which activate vir genes responsible for synthesizing single-stranded T-DNA copies [161,162]. These T-DNA fragments traverse host barriers and enter the plant nucleus, where they integrate into the host genome. Beyond plant transformation, mobile genetic elements (MGEs) have also been observed to transfer from plants to microbes, expanding the complexity of interkingdom gene flow [163,164,165,166]. Within bacterial communities, HGT represents a critical mechanism for disseminating MGEs in the rhizosphere, facilitating adaptive traits such as antibiotic and heavy metal resistance [167,168,169,170,171]. The proliferation of soil contamination by antibiotics and heavy metals is increasingly attributable to heightened anthropogenic activities [172]. The dissemination of resistance genes is crucial for bacterial adaptation and survival in contaminated rhizospheres. Furthermore, MGEs are integral in modulating microbe–plant interactions. In the case of *Pseudomonas fluorescens* Pf-5, numerous MGEs within its genome have been identified as pertinent to survival in natural environments and to its pathogenic interactions with crops. Notable examples of pathogenic genes include effector proteins and enzymes that modify cell walls, facilitating plant infection [173].

Horizontal gene transfer (HGT) significantly contributes to the acquisition of genes associated with bacterial functions that provide advantages to plants. Instances of advantageous roles played by bacteria concerning plants encompass the solubilization of phosphate, the production of antimicrobial substances, the suppression of ethylene biosynthesis, the promotion of systemic disease resistance, and the generation of plant growth hormones [174]. Comparative analyses of various biological entities, including animal pathogens, phytopathogens, saprophytes, endophytes/symbionts, and PGPRs, reveal that animal pathogens possess the fewest genes linked to plant-beneficial traits, particularly genes advantageous to plant hosts. PGPRs exhibit the highest gene counts for such traits, aligning with their strong association with plants, whereas animal pathogens show the lowest plant affinity [174]. Notably, twenty-three genes associated with these beneficial plant functions were carefully curated for gene acquisition and loss analysis. Evolutionary investigations suggest these genes were acquired across diverse taxa. For example, 18 horizontal gene acquisition events were linked to nifHDK genes associated with nitrogen fixation in proteobacterial PGPRs, and 21 events related to acds, which encodes 1-aminocyclopropane-1-carboxylate (ACC) deaminase. These transfers are thought to have contributed to the unique gene combinations seen in PGPRs [174]. Despite these insights, significant knowledge gaps remain concerning the ecological and evolutionary drivers of HGT in plant-associated microbiomes. The influence of specific environmental cues, microbial community structures, and host pressures on HGT remains insufficiently characterized. Furthermore, the functional integration and regulatory stability of acquired genes are poorly understood. Thus, future research should focus on unraveling the regulatory and environmental determinants of HGT, especially under field-relevant conditions. Additionally, tracking real-time gene flow and determining the fitness impact of acquired traits in complex microbiomes remain critical areas requiring urgent attention.

In summary, ecological predictability remains low because interacting abiotic drivers, priority effects, niche competition, functional redundancy, host genotype and ontogeny, and climate-linked pathogen pressure dissociate taxonomy from function. Progress requires longitudinal, multi-factor field and mesocosm studies that manipulate pH, temperature, aeration, and moisture, track pathogen–microbiome and host and integrate in situ sensing with genome-resolved meta-omics and trait assays. These designs will enable mechanism-based models that forecast resilience and disease risk across environments and genotypes, narrowing the gap between the laboratory and the field.

## 4. Biological Challenges

### 4.1. Limited Functional Characterization of Microbiome Members

Although thousands of microbial species linked to plants have been cataloged using recent developments in DNA sequencing, many of these organisms’ functional diversities are still mostly unknown. For uncommon, unculturable, or uncharacterized microorganisms that might be crucial to plant health, this “function blind spot” is especially noticeable. Although they offer valuable insights, predictive bioinformatics tools like functional annotation using KEGG or COG databases frequently lack the clarity or accuracy required to draw predictions that may be put into practice. Furthermore, regulatory variations or environmental limitations may prevent the in situ expression of microbial functions deduced from sequence homology [175]. Linking microbial genes to phenotypes in field settings, where many pressures and community dynamics are present, is also extremely challenging. Integrating many “omics” technologies will be necessary to address this, including metabolomics to evaluate the biochemical outputs of microbial activity [176], metaproteomics to examine proteins that are actively being created [177], and metatranscriptomics to record patterns of gene expression. Functional tests utilizing sophisticated microscopy, microfluidics [178], and synthetic microbial communities (SynComs) will also be crucial for confirming theories in controlled settings before field testing [179]. Enhancing cultivation techniques for “microbial dark matter”, that is, the sizable portion of microorganisms that are resistant to conventional culturing, can also greatly increase our ability to assess and utilize microbial functioning.

### 4.2. Host-Specificity and Environmental Context Dependency of Microbiome Assemblies

Numerous internal and external variables influence the extremely specialized interactions that occur between plants and the microbiomes cohabiting with them. A key factor in defining the makeup and activity of microbial communities is host genotype. For instance, the root exudates produced by various cultivars of the same crop might differ greatly, drawing distinct microbial populations. These exudates serve as specific filters for microbial colonization since they are made up of sugars, organic acids, amino acids, and secondary metabolites [180]. Plant developmental phases, such as seedling, blooming, or senescence, also change the physiological state of the plant, which affects the persistence and recruitment of microorganisms [181]. It is challenging to generalize microbiome activities across places or time periods due to the additional layers of complexity added by environmental parameters such as temperature, moisture, pH, nutrient levels and soil structure [182]. Because of this, biostimulants or microbial inoculants created for one crop or situation sometimes do not work for another [183]. Designing robust and adaptable microbiome-based therapies requires an understanding of the molecular processes underpinning this context reliance. In conjunction with genome-wide association studies (GWAS) and systems biology techniques, sophisticated experimental designs utilizing a variety of genotypes and settings will aid in identifying important host characteristics and microbial factors that control compatibility and efficacy.

### 4.3. Achieving Stability, Resilience and Predictability of Engineered Microbiomes

Engineering or modifying microbial communities to stimulate plant growth, improve stress tolerance, or inhibit disease is one of the ultimate objectives of plant microbiome research. But creating durable and stable microbiome topologies is still a major biological challenge. Whether as synthetic consortia or single strains, introduced beneficial microorganisms frequently either fail to colonize the plant or are rapidly outcompeted by local bacteria, making the inoculation useless [184]. However, a fundamental knowledge gap remains in our understanding of the ecological processes that govern microbiome assembly, particularly the roles of host-mediated selection, niche complementarity, and priority effects, which influence both the timing and sequence of microbial colonization. Furthermore, microbiome stability can be upset by environmental disturbances like drought, pesticide use, or nutrient additions, which can change the makeup and function of communities in unforeseen ways [185].

Identifying reliable microbial candidates may be aided by identifying “core microbiomes” that are consistently associated with specific plants or habitats. To find out if early invaders might serve as keystone species that influence the long-term trajectories of the microbiome, longitudinal field research and modeling of microbial succession dynamics are required. Furthermore, new prebiotic techniques (like targeted plant exudation) and distribution methods (like seed coatings, soil supplements, or foliar sprays) can improve the establishment and permanence of advantageous microorganisms. The difficulty is in accurately forecasting and managing these results for commercial implementation.

### 4.4. Exploring the Co-Evolution and Domestication Effects on Plant–Microbe Interactions

Over millions of years, interactions between plants and microbes have coevolved, and natural selection has refined many microbial features in response to plant cues and vice versa [186]. Mycorrhizal fungi and vascular plants or legumes and nitrogen-fixing rhizobia are examples of symbiotic interactions that result from long-term co-evolutionary processes that have given both partners adaptive advantages [187]. These delicately balanced connections, however, could have been upset by contemporary agricultural methods, including monocultures, chemical inputs, and selective breeding. Research indicates that, compared to their wild counterparts, domesticated crops often possess less diverse or less beneficial microbiomes [188]. It is possible that during domestication and breeding for yield or stress tolerance, traits crucial for microbiome recruitment or compatibility, such as root architecture, exudate composition, and immunological signaling, were unintentionally lost or downregulated [189]. Beneficial plant–microbe connections may be revived by reintroducing or restoring such features into elite cultivars. By using comparative genomics, phylogenetics, and paleogenomics to understand the evolutionary paths of both plants and the microorganisms that are associated with them, important interactions that have influenced plant fitness in the past and may be reactivated or designed for use in agriculture in the future may be identified. Important considerations concerning how microbiomes will respond or not to the swift changes in temperature, land use, and ecosystem degradation are also brought up by this co-evolutionary viewpoint.

Therefore, plant microbiome research will need to change from descriptive to interventional and predictive science during the next ten years. Ecology, molecular biology, computer modeling, and agricultural sciences must all be used to develop integrated, multidisciplinary solutions to the biological problems mentioned above. To fully realize the promise of plant-associated microbiomes, a thorough mechanistic knowledge of microbial activities, host-specificity, ecological dynamics, and evolutionary history will be essential. In addition to improving our fundamental knowledge of plant biology, overcoming these obstacles will open the door for microbiome-based approaches to address global issues in sustainable agriculture, food security, and climate resilience. The final frontier is still biological complexity, despite the instruments’ fast advancement.

Key bottlenecks include poor in situ functional insight for many taxa, strong dependence on host and environment, weak persistence of introduced strains or consortia, and loss of plant traits that harbor helpful microbes. A practical way forward is to combine metabolomics, metaproteomics, and metatranscriptomics with focused assays such as advanced microscopy, microfluidics, and SynCom tests to connect genes with phenotypes. In parallel, map host drivers across genotypes and environments using GWAS and systems biology to explain when and where assemblies succeed. Design consortia that exploit priority effects and niche complementarity, deliver them through seed coatings or targeted exudation, and confirm performance in long, field-based trials. Use comparative genomics, phylogenetics, and paleogenomics to recover or reintroduce lost recruitment traits in elite cultivars. Together, these steps will shift the field from description to prediction and real-world use.

## 5. Agricultural and Applied Challenges

### 5.1. Translating Research to Field Applications

Translating laboratory research findings into field applications in agricultural production has received considerable attention from scientists, policymakers, and manufacturers. In the past ten years, the development of microbiome products as biopesticides or biofertilizers, market access, and applicability have recently increased due to advances in studying plant-associated microorganisms. Still, there have been many challenges involved in translating research to field applications, specifically in the context of microbial inoculant applications, including low efficacy and uneven performance under field conditions [190,191]. Though the use of these products in field conditions is in its infancy, successful potential results from using microbial inoculants have been reported. In Brazil, soybeans inoculated with Nitrogen-fixing *Bradyrhizobium* spp. eliminated the need for Nitrogen fertilizers, which significantly reduced the emission of greenhouse gases, 78% increased yield, and yearly cost savings of US$13 billion [192]. Despite the success of applying microbial-based inoculants under field conditions, some complexities are associated with research translation and the scaling of microbial inoculants under field conditions. These challenges would give a clearer insight into their efficacy across different field conditions and agricultural settings.

Key factors affecting the translation of laboratory findings to the field include inoculant formulation, application methods, crop cultivar, and interaction with resident microbial strains in the soil [191]. Thilakarathna and Raizada [193] documented meta-analyses of 28 peer-reviewed studies that presented extensive inconsistency in the effectiveness of various *Sinorhizobium* and *Bradyrhizobium* species under field conditions. These microbial inoculants differed in their efficiency for grain-N yield (−6% to +176%) and grain yield (−34% to +109%) equated with uninoculated controls. Inconsistency was attributed to diverse factors such as soybean x rhizobia strain interactions, soybean genotype, formulation, inoculant titre, application technique, and weak persistence of rhizobia strains in soil.

Data management and sharing difficulties in agricultural research have also been reported. Scientists are frequently faced with problems in standardizing data formats and sharing research findings efficiently, which can hinder the use of valuable datasets [190]. Despite the variety of microbial inoculant products reported to have the ability to solubilize phosphorus (P), there is no clear agreement among researchers on the advantages of P-solubilizing microorganisms (PSM) for plant production. Many studies documenting the benefits of PSM application were performed under controlled environments. Whether microbial processes for P solubilization shown in vitro study would occur in the field soil at a level important for plant nutrition is debatable. Studies under controlled laboratory and field conditions have not been able to prove a well-defined relationship between increased P in the soil by PSM and crop yield. Raymond et al. [194] stated that the approach of inoculating PSM into non-native soil ecosystems to gain an additional solubilized P for plant uptake is unable to prove that it is a dependable strategy to boost crop P nutrition under field conditions and concluded that the method was not a fact.

The challenge of knowledge integration from different disciplines is a barrier that is deepened by biases due to the invisibility of most microorganisms. Hence, they are unfamiliar to most individuals, so the prospects they offer to solve and prevent problems efficiently are frequently unexploited by society and decision-makers, with the adverse aftermath that follows. To correct this problem of lack of crucial knowledge, organizations like the International Microbiology Literacy Initiative (the IMiLI) could recruit, teach, and make available teaching resources relevant to society as significant tools that unveil the potential impacts of microorganisms and their activities in our lives and environments. Also, their societal needs and relevance for decision-making and acceptance at all levels [195]. This approach would increase the need for collaborations across diverse interdisciplinary fields by addressing many professional biases and integrating various research knowledge and strategies [196]. Furtherance to implementing field trials to validate laboratory findings creates logistical difficulties, including obtaining consent from local farmers and stakeholders for soil sampling, application of microbial inoculants, and comprehension of their decision-making approaches. According to Gourlay et al. [197], field trials are key to assessing the practical applicability of laboratory research discoveries. Nonetheless, regulatory interferences and insufficient engagement with local farming communities may influence the implementation of field trials [196,197].

In addition, other reasons that explain the gradual adoption of innovative technological methods, such as microbial inoculants in field practice, include several other factors, like their complexity, the cost involved and farmers being trained to acquire the expertise. In addition, some precision agricultural information and skills have been extensively embraced, while other aspects have been delayed due to apparent risks and insufficient training about their advantages [198,199]. In many countries, such as New Zealand and China, strains of rhizobia have been intensively studied and cultured to the extent that the bacteria have been applied to symbiotic legume seed and sown on rhizobium barren soil, which infected the root hairs of developing clover plants and resulted in the nitrogen transformation in the soil to plant nutrients within clovers and other leguminous plants. Despite this, many technical and physical challenges are linked to the effective delivery of inoculated legume seeds, including proper seed coating. For example, seed coating was developed to protect sensitive *Rhizobium* nitrogen-fixing bacteria because most seeds contain natural toxins against soil deterioration, which also destroy rhizobia. Therefore, to provide precision, finely milled nutrients are placed right where seeds are planted in or on infertile soil. Hence, nutrients are supplied to the seedlings, not to the soil [200]. Without proper training and skills, farmers and stakeholders would be unable to implement the novel technological approach and strategy in the field.

Moreover, scaling up microbial inoculants and validating their efficacy in field trials in diverse agrarian settings presents a significant challenge. For example, factors like soil type, environmental conditions, and local farming practices have been reported to substantially impact the implementation of these microbial inoculants in field experiments [201]. Building trust and collaboration also affects the successful scaling of microbial inoculants, which requires creating understanding and reliability among stakeholders, farmers, and agriculturalists. According to O’Callaghan, Ballard and Wright [191], stakeholders may express concern regarding data privacy, as the potential misuse of shared information can significantly impede the collaborative frameworks necessary for the successful adoption and large-scale implementation of microbial inoculants in agricultural systems. However, developing and establishing strong support networks and offering comprehensive training can help alleviate these concerns [196,197].

To effectively scale up microbial inoculants in various agrarian environments, it is important to address the problems linked with executing field experiments, data management, adopting innovative technologies, and stakeholder connection information [190]. Exploiting advanced monitoring and feedback systems that provide relevant and up-to-date information on soil type, soil health status, and microbial activity can improve decision-making for farmers employing microbial inoculants in their farms [202]. Farmers can employ these systems to comprehend the impact of their approaches, which would help them adjust accordingly, consequently boosting the effectiveness of microbial inoculant applications [191,203].

### 5.2. Regulatory and Ethical Considerations in Microbiome-Based Products

The successful navigation of the regulatory framework and dealing with the complexity and critical ethical considerations are essential for the regulation, effective manipulation, and implementation of microbiome-based agricultural products for commercial use [204]. In general, the regulatory frameworks concerning microbiome-based products are different across regions and influenced by the intended use of the products. In the United States of America, these products are classified as either food, dietary supplements, or drugs, all of which have distinct regulatory pathways [205]. For instance, in the United States of America, many microbial inoculants, often referred to as biofertilizers or biopesticides, have been approved for diverse agricultural purposes [206]. The United States Department of Agriculture (USDA) and the Environmental Protection Agency (EPA) regulate these microbial inoculants; some of these products and their applications are highlighted in Table 1. Although these products are approved for agricultural use, their efficiency can differ depending on application methods, specific crop-pathogen interactions, and environmental conditions.

The challenge of classifying some of these microbial products properly is one of the major problems. The distinctions in regulations can lead to misperceptions among product developers regarding which recommendations apply to their products [221,222]. For example, some of these products may be advertised or sold as soil probiotics or microbiome inoculants. However, certain bioproducts could be subjected to stricter drug regulations based on their marketed benefits and intended usage [223]. Likewise, ensuring safety and efficacy standards is paramount, specifically given the possibility of adverse effects from manipulating microbiomes.

In this view, regulatory agencies necessitate comprehensive information about the microbial species, their mechanism of interaction within the environments, and how they impact health and ecosystems over time [224]. Hence, it demands a prominent framework for pre-market risk assessments and post-market surveillance to monitor continuous product function and safety [225]. Microbiome research frequently deals with confidential personal information, and problems surrounding data ownership, control, accessibility, and sharing pose substantial regulatory challenges in implementing microbial inoculants in field trials [225,226]. The scientific community continues to debate the patentability of microbial strains extracted from various samples and how genetic modifications should be regulated appropriately [227].

Furthermore, the ethical consequences of manipulating microbiomes for commercial uses in soil increase concerns about introducing non-resident microbiomes, loss of biodiversity, and the balance in the ecosystem [228]. Relevant studies have shown that microbial inoculants improve plant productivity by advancing crop resilience [221,229]. It may also potentially disturb long-established microbial communities in the ecosystems that have co-evolved alongside plants, with implications for long-term processes [230].

Researchers are encouraged to be transparent and ethically communicate with stakeholder groups, including farmers and consumers, which is greatly recommended through public engagement, social media management, and an active website [231]. Joining public forums for scientific discourse, employing Google Analytics, and requesting feedback from participants in joining public engagement activities are essential in strategically improving and deploying microbiome technologies ethically [232,233]. Therefore, educating the public about the benefits and potential risks associated with manipulating the microbiome is essential to fostering acceptability and trust [234]. Additionally, ethical considerations should be in place regarding equitable access to microbiome-based innovations [235]. Ensuring that smallholder farmers take advantage of these innovative technologies without facing complexities or exorbitant costs is critical for promoting sustainable agricultural practices worldwide [236]. Also, careful consideration of the short and long-term ecological effects of introducing engineered microbiomes into agricultural systems is important [237].

Critical consideration of ethical frameworks that should guide studies and ensure microbial interventions used in farm practices do not result in unintentional after-effects [235] that could cause a decrease in genetic diversity within crops, and crucial native microbes or harm ecosystems [238]. A collaborative strategy that involves scientists, industry stakeholders, regulators, and the public is crucial to addressing these challenges efficiently [239], thus fostering innovative agricultural biotechnology, short and long-term ecological sustainability, biosecurity, and economic productivity [228].

### 5.3. Economic Viability of Microbiome-Based Interventions in Agriculture

For the successful adoption of these innovative microbiome-based interventions in agricultural practices [239], assessing the economic feasibility of microbiome-based interventions in agriculture is a critical step toward the successful application of microbial inoculant solutions in farm soils [235]. This comprises estimating the cost-effectiveness of these microbial interventions [206] and addressing the economic difficulties that small stakeholders and farmers encounter [222]. Microbiome-based interventions, like microbial inoculants, have shown the potential to enhance crop resilience and increase productivity [206]. Current research findings have documented that microbial-based products are cost-effective and ultimately advance soil fertility, enhance nutrient uptake, improve plant health, and substantially boost crop yields [240,241,242], thus addressing the problems of modern agriculture [242].

Nevertheless, the economic viability of microbial inoculants in agricultural soils depends on indicating a well-defined return on investment (ROI) for stakeholders and farmers, which requires comprehensive data on performance across several conditions [243]. Additionally, to establish the cost-effectiveness of microbiome-based products, it is imperative to compare them with traditional agricultural inputs such as pesticides and chemical-based fertilizers [244]. Experiments with many microbial inoculants have established their valuable role in plant growth through active root colonization and stimulation of plant growth support mechanisms, compared to chemical-based products [244,245].

Although initial costs for microbiome-based products may be high. However, their long-term benefits and agronomical effects [246], like decreased usage of chemical products, environmental sustainability, enhanced soil health, and human health, could offset these prices [247]. Inoculating microbiome-based products in agricultural soil can contribute to long-term agricultural sustainability and agroecological benefits by reducing the dependency on artificial chemicals and advancing the biodiversity of the soil ecosystem [244,246]. Over time, the agricultural sustainability aspect can result in cost savings, specifically in terms of soil health maintenance and reduced cost of environmental remediation [248].

In addition, the high initial investment linked with these products is a major economic barrier to targeted consumers’ extensive adoption and use of microbiome-based interventive products [249]. Especially, many small stakeholders may not have the upfront investment capital in technological innovations without clear evidence of their value [190]. Therefore, mechanisms for financial support, like grants or subsidies from non-governmental organizations, government agencies, and farmer organizations, could help address this economic barrier [250]. The introduction of various effective market access and distribution channels is pivotal to ensuring that farmers have reliable access to these microbiome-based products [251]. The lack of established market access and supply chains can delay availability and increase the prices of microbiome-based products [252]. Effective collaborations between the manufacturers and local distributors can address the multi-prolong challenges farmers and other stakeholders are currently facing, advance market access, and lessen logistical problems [250,253].

Furthermore, farmers and stakeholders need to be trained regularly on the advantages and application techniques of microbiome-based products, which will boost their confidence in applying them efficiently [252]. Training programs that prove effective case studies and deliver hands-on knowledge can expedite adoption [251]. Moreover, skepticism about these interventions must be overcome by increasing awareness of the long-term economic, environmental, and health benefits [250]. Reportedly, supportive regulatory environments and policies have been proven to advance innovation and lessen the barriers manufacturers encounter to market access for microbiome-based products [252]. Clear regulatory frameworks on labeling, safety standards, and efficacy of products can boost consumer trust and inspire swift adoption among agriculturalists [254]. Policymakers should work towards setting up guidelines that accelerate research funding, support product development, and ensure efficacy and safety [255]. Consequently, farmers and stakeholders can improve the acceptance of these potential agricultural solutions by developing an environment that supports novelty through financial assistance, training, education, and clear regulations [254].

Field translation is limited by variable inoculant performance, formulation and delivery constraints, uneven data standards, low farmer readiness, regulatory ambiguity, and unclear ROI and market access. A workable plan is to run factorial trials across sites with shared metadata standards, strengthen formulations and delivery through encapsulation and seed coatings verified in long-term studies, add decision support from in situ monitoring, and involve growers early to align practices and training. Regulatory clarity with pre-and post-market risk frameworks, data governance, and transparent efficacy labeling will build trust, while ethical guidance should safeguard biodiversity and equitable access. Finally, targeted financing (grants, subsidies), reliable distribution channels, and extension programs that demonstrate cost–benefit against conventional inputs can reduce adoption risk and improve ROI across contexts.

## 6. Future Directions and Emerging Opportunities

**Figure 3:**
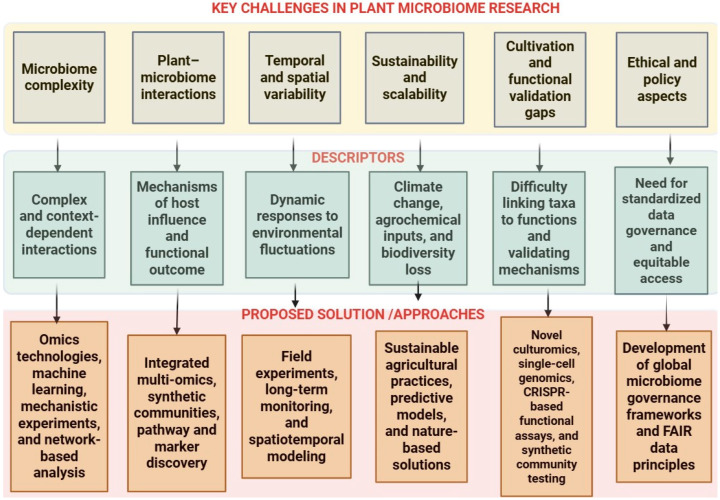
Structured overview of challenges, mechanistic descriptors, and proposed interventions in plant microbiome research.

Future research in plant microbiome science is poised to leverage advanced methodologies, providing new pathways to address key agricultural challenges and promote sustainable crop production. Figure 3 provides a structured overview of identified challenges, mechanistic descriptors, and proposed interventions in plant microbiome research. One prominent future direction involves multi-omics integration, which combines genomics, transcriptomics, proteomics, and metabolomics to capture a comprehensive profile of microbial communities and their interactions within plant hosts. This approach can clarify how different microbial communities function together and contribute to plant resilience, growth, and nutrient acquisition [4]. The development of high-throughput sequencing and improved data integration tools allows for better exploration of plant–microbiome relationships in various environmental contexts, aiding in the identification of microbial functions that directly support plant health [1]. However, scaling multi-omics approaches to field applications requires advanced computational pipelines and bioinformatics tools that can handle and interpret complex data volumes efficiently [256].

In parallel, synthetic biology holds promise for creating engineered microbial communities tailored to enhance specific plant traits. By designing microbial consortia with optimized functional pathways, researchers aim to improve crop tolerance to abiotic and biotic stresses, such as drought and pathogen pressure [2]. Synthetic biology tools, such as CRISPR, allow for precise editing of microbial genomes, enabling scientists to modulate gene functions within microbial communities that can lead to optimized plant growth outcomes [9,12]. This direction, while transformative, must overcome ethical and regulatory barriers to ensure safe deployment and environmental compatibility of engineered microbes [14].

Another promising avenue is the development of predictive models using machine learning (ML) and artificial intelligence (AI), which can process complex, multi-dimensional data and predict plant–microbiome interactions under various environmental scenarios [8]. These models can identify key microbial taxa and functions that support plant health, enabling targeted interventions in agricultural systems. For example, ML algorithms could predict which microbial inoculants will be most effective in specific soil types or climates, thereby improving the effectiveness of biofertilizers and biocontrol agents [13]. Yet, implementing such models in the field necessitates robust and validated datasets that represent diverse ecosystems and cropping systems [257].

Microbiome engineering and microbial inoculant formulation are gaining attention as methods to introduce beneficial microbial strains directly into agricultural soils or plant root zones. These formulations are designed to strengthen soil health, nutrient availability, and pest resistance [65,258]. Despite significant progress, the practical application of these inoculants on a large scale requires more efficient formulation techniques, as well as solutions to address challenges related to product stability and scalability [13,259]. Additionally, the implementation of microbial-based solutions should be accompanied by a regulatory framework that ensures the safety and efficacy of such interventions, especially given their potential environmental impact [260].

Also, policy and cross-disciplinary collaboration represent crucial future steps in translating plant microbiome research into practical agricultural solutions. Collaborative efforts between agronomists, microbiologists, soil scientists, and policymakers are essential for creating guidelines and incentives that encourage sustainable microbiome management practices [35]. Government support for microbiome research initiatives and funding for microbiome-based technologies would accelerate the commercial development of microbial products and promote global agricultural sustainability [261]. Additionally, incorporating farmers’ knowledge and experience can aid in designing user-friendly and effective microbial products suited to real-world agricultural practices [13].

## 7. Conclusions

Taken together, plant microbiomes hold immense promise for revolutionizing agriculture by enhancing crop productivity, resilience, and sustainability, despite global challenges such as climate change and food insecurity. However, significant hurdles remain, including technical constraints in sequencing with standardization and reproducibility, data integration and analysis, the limited functional characterization of microbiome members, spatiotemporal variability, host specificity and environmental context dependence, the need to achieve stability, resilience, and predictability in engineered microbiomes, co-evolution and domestication effects, plant genotypes’ influence, and the challenge of translating laboratory-based discoveries into field-ready applications. In parallel, agricultural deployment must address regulatory and ethical requirements and demonstrate economic viability for end users. Addressing these challenges will require interdisciplinary approaches that integrate multi-omics technologies, advanced bioinformatics, and artificial intelligence to unravel microbial functions and dynamics. Furthermore, fostering international collaboration and creating inclusive frameworks will be essential to ensure the equitable distribution of microbiome-based technologies globally. Future research should prioritize the development of robust predictive models for microbiome manipulation, leverage synthetic biology to engineer beneficial plant–microbe interactions, and explore the regulatory and ethical implications of these emerging interventions. Moreover, there is a need to establish transparent reporting, shared metadata, and public benchmarks to enable comparability across sites and seasons, and to link models to decision metrics relevant to agronomy. Additionally, long-term field studies are necessary to validate microbiome-based solutions under diverse environmental and agricultural conditions. By targeting these challenges, technical, biological, and applied, plant microbiome research can unlock its full potential, contributing to resilient and sustainable agricultural systems capable of feeding a growing global population.

## Figures and Tables

**Table 1:** Widely used live microbial inoculants for disease and pest control and growth promotion in plants.

Product Name	Company Name	Microbial Species	Uses	References
RootShield	BioWorks, Victor, NY, USA	*Trichoderma harzianum*	Control plant diseases and growth promotion	DeGenring and Poleatewich [207,208]
N-Dure	Verdesian Life Sciences, Cary, NC, USA	Various *Bradyrhizobium* and *Rhizobium* strains	Used as bioinoculants for legumes to improve nitrogen fixation	Braun, et al. [209]
XenTari	Biofa AG, Münsingen, Germany	*Bacillus thuringiensis*	Different strains of *Bacillus thuringiensis* are employed as pesticides	Arthurs and Dara [210], Stein et al. [211]
Dipel	Valent BioSciences, Libertyville, IL, USA	*Bacillus thuringiensis*	Widely used as biopesticides	Radwan and Taha [212]
BlightBan A506	Nufarm, Melbourne, Australia	*Pseudomonas fluorescens*	Control phytopathogens and plant growth promotion	Stockwell, et al. [213]
Serenade	Bayer, Leverkusen, Germany	*Bacillus subtilis*	Suppress plant disease and boost plant growth	Becker, et al. [214]
Actinovate	Novozymes, Bagsværd, Denmark	*Streptomyces lydicus*	Manage diseases in a variety of crops	Marine, et al. [215,216]
BotaniGard	BioWorks, Victor, NY, USA	*Beauveria bassiana*	Entomopathogenic fungus used for insect management	Karise, et al. [217]
Clariva	Syngenta, Basel, Switzerland	*Pasteuria nishizawae*	*P. nishizawae* spores are used to control nematode	Jensen, et al. [218]
Double Nickel 55	Certis USA, Columbia, MD, USA	*Bacillus amyloliquefaciens*	Used to control plant diseases and improve plant growth	Pethybridge, et al. [219]
Met52	Novozymes, Bagsværd, Denmark	*Metarhizium anisopliae*	Entomopathogenic fungus employed for insect control	Sullivan, et al. [220]

## Data Availability

No new data were created or analyzed in this study. Data sharing is not applicable to this article.
